# Spontaneous preferences and core tastes: embodied musical personality and dynamics of interaction in a pedagogical method of improvisation

**DOI:** 10.3389/fpsyg.2015.00522

**Published:** 2015-05-21

**Authors:** Julien Laroche, Ilan Kaddouch

**Affiliations:** R&D Laboratory, Akoustic ArtsParis, France

**Keywords:** free improvisation, personality, music pedagogy, embodied music cognition, coordination dynamics, social interactions, preferences, tastes

Free improvisations are unprecedented and underdetermined: their content and the way they unfold are not known in advance. Improvised performances have to be actively shaped over time. To do so, the improviser must articulate his embodied experience. In other words, he has to make sense of his experience^1^ by relying on his bodily know-how. And so this process of improvisation *personally* involves the improviser. Therefore, it should be a relevant point of entry for the study of his musical “personality” (or “identity,” see Hargreaves et al., 2002). Firstly, to put this argument forward, we link free improvisations and personality in the light of embodied (enactive) and dynamical approaches. However, free improvisations are often played as a group. Secondly, so as to link improvisations and personality in the context of human interactions, we present a pedagogical method of free improvisation (the Kaddouch pedagogy; Kaddouch and Miravète, 2012) in which interactivity between learner and teacher is a core aspect. Then, we will show that the process of interaction by itself allows to unveil, to foster and to expand both improvisational skills and musical personality. Finally, we propose theoretical bases for this method, plus some explanations dealing with its effects.

## Personality and improvisation as embodied, dynamical, and social phenomena

Traditionally, cognitive sciences assume that behaviors are caused by mental, computational, and individual processes. However, this view has been challenged by new perspectives. First, embodied accounts such as the enactive approach have pointed out that, by interacting with the world, our active *living* body and our *lived* experiences co-constitute each other. In other words, our experiences guide and emerge from what we do (Varela et al., 1991). Traditionally, personality also refers to abstract and mental characteristics (e.g., traits) that are supposed to underlie behavior (Goldberg, 1990). In this context, personality is an attribution based on the observation of a person's *living* behavior. Yet, personality is also *lived* from the bodily perspective of its holder (Fuchs, 2012): it is mainly from our own perspective that the enactment of our behavior makes sense. A full, embodied account of personality thus requires to link a *lived* experience with its observable *living* expression.

Embodiment is also central in the constitution of musical experiences^2^ (Krueger, 2011a,b; Schiavio, 2012), and movements are a relevant way to probe these experiences (Leman, 2007). In particular, free improvisations directly involve the performer's personal embodied experience (Iyer, 2002). The path he is taking during the performance means something *for* him; it makes sense from his own perspective. At the same time, the externalization of his embodied experience leaves a trace that can be analyzed: the path of his performance also means something *about* him. Free improvisation is therefore a mean to reveal oneself from both a personal *lived* perspective, and the perspective of the observer of one's *living* behavior. By linking these two perspectives, free improvisations constitute a relevant starting point for the embodied exploration of musical personality.

As a coupling between experience and activity, embodiment is a dynamical process (Laroche et al., 2014). Dynamical approaches show how complex patterns coordinate and evolve over time (Kelso, 1995; Van Orden et al., 2003). Thereby, they provide concepts and tools able to link lived experience and observable living activity (Varela, 1999; Froese and Fuchs, 2012). Musical experiences (Large, 2008, 2010a,b), musical performances (Demos et al., 2014), and musical improvisations (Pressing, 1988; Borgo, 2005) have been discussed and modeled in such terms. During an improvisation, the embodied dynamics of the improviser is simultaneous to the dynamics of the musical trace he is leaving. Analyzing the dynamics of his performance should therefore shed light on the coupling between his lived experience and his observable activity.

The emergence and the manifestation of personality have also been accounted in dynamical terms (Lewis, 1997; Vallacher et al., 2002; Nowak et al., 2005). From this perspective, personality can be thought of as a set of dynamical *tendencies* that shape our spontaneous behaviors. The notion of dynamical tendencies has the advantage of encompassing both the stability and the variability of behaviors in a single explanatory framework (Ninot and Fortes, 2007). The organization of musical tendencies, in terms of performances' stability and variability, should thus shed light on the performer's embodied musical personality (Hargreaves et al., 2002; Van Vugt et al., 2013).

In the end, since embodiment is constituted in our interactions with the world, our behaviors and our experiences are directly shaped by the dynamics of our interactions with others (De Jaegher et al., 2010; Froese et al., 2014; Laroche et al., 2014). This has been acknowledged in the fields of personality (Kyselo, 2014), music experiences (Krueger, 2013; Moran, 2014), and improvisation (Gratier and Magnier, 2012; Schober and Spiro, 2014). Interactions with others should therefore play a role in one's embodied musical personality. To asset this hypothesis, we are now presenting a pedagogical method of free interactive improvisation. This method will be illustrated with two concepts that emerged from practice (spontaneous preferences and core tastes), and which will be explained theoretically.

## Spontaneous achievement zones and spontaneous preferences

Pedagogy of improvisation has been discussed by Kratus (1995). According to his opinion, the teacher plays an observational and instructional role. In our method, however, teacher and learner interact as they improvise at the same time on the same piano. As we will see, the teacher uses the process of interaction to foster learning, and to explore musical personality.

Although free improvisations are unprecedented and underdetermined, we observe recurrent patterns of expression (rhythms, gestures, tonality, harmony, melodic form…). These patterns are easily played, without any instruction to enact them, pointing out the existence of “spontaneous achievement zones” (i.e., privileged regions of behavior among the full space of behavioral possibilities). Such spontaneous preferences are specific to each learner, even at a very young age and with a low level of expertise (Kaddouch and Laroche, 2014): they constitute an embodied signature of their musical personality.

Yet, in order to manage the improvisation of the learner, the teacher has to help him to discover his spontaneous achievement zones. To do so, the teacher probes the learner's spontaneous tendencies by testing the stability of the latter's patterns during the interaction. The teacher steadies these patterns by coordinating with them, thereby enabling the learner to consolidate and develop them. In this manner, interaction enables the teacher to unveil and foster learners' embodied musical personality.

Behavior can be explained dynamically by the coupling of its component processes (Bernstein, 1967; Kelso, 1995). The self-organized interactions of these components constitute the intrinsic dynamics of an embodied subject. Some patterns of interactions are more stable than others: this gives rise to a dynamical landscape, meaning, the regions of attraction that orientate behavioral trajectories (Kelso, 2009). In a laboratory settings, the topology of this landscape is probed by testing the stability of various patterns of coordination. As this dynamical landscape differs among individuals and as it is modified as a whole by learning (Kostrubiec et al., 2012), it constitutes a dynamical signature of the subject's embodiment; it reflects his habitual way of inhabiting the world, and the past experiences that strengthen his intrinsic dynamics. This explains what we coined as spontaneous achievement zones. Similarly, the self-organization of embodied interactions between persons gives rise to dynamics that are properly collective (Oullier and Kelso, 2009). These dynamics coordinate the behaviors of interacting subjects (Laroche et al., 2014). The learner can thus rely on the dynamics of interaction to coordinate his own movements with less difficulty and fewer efforts. Interacting with the teacher therefore strengthens the learner's patterns. In this case, learning consists in stabilizing the intrinsic dynamics of one's embodied musical personality.

Spontaneous behavioral tendencies are said to be “preferred” (Kelso, 2012). However, from a lived perspective, are these preferences “attractive” on top of being “attracting” ? If behavior is biased by spontaneous preferences, this might prevent learners to develop their improvisation with new patterns that they might desire to enact with a vengeance. Moreover, learners get easily bored with their own spontaneous achievement zones. Kelso and Engstrom (2006, p. 222) see exploration as a complementary aspect of preferences. We now shall see how interacting can help to explore other behavioral possibilities, in order to expand learning and revealing deeper aspects of musical personality.

## Escaping from spontaneous preferences to fulfill core tastes

Instead of matching the learner's patterns, the teacher can alternatively propose a different one. In this case, even if the current pattern of the learner is well executed, it now sounds wrong in the interpersonal context, and so does it from the perspective of his lived experience. In order to continue communicating and improvising in a coherent and pleasant way, the learner must reorganize his behavior in a flexible way so as to fit the constraints of collective performance. Doing so, he has to explore some patterns outside of his spontaneous achievement zones. Usually, this enhances the fluctuations of his performance, until he suddenly jumps toward a new pattern that simultaneously makes sense for him, and fits the collective performance. Here, the process of interaction enables to explore some more aspects of the learner's personality hinder by his spontaneous tendencies. It allows the learner to fulfill what we coined as “core tastes,” that is, patterns of behavior that entail experiences he wishes to live^3^, but that he cannot coordinate all by himself (Laroche and Kaddouch, 2014).

By blocking the learner's pattern with a different one, the teacher causes a loss of stability in the interpersonal pattern of coordination. Because collective dynamics constrain individual behaviors by attracting them toward interpersonal patterns, the strength of the learner's spontaneous attractors gets weakened. In the ongoing interpersonal context, they even get “repelling.” The dismantlement of previously formed patterns and fluctuations of activity are very important for the emergence of new patterns of coordination (Kelso, 1995). Consequently, behavioral attraction migrates from the learner's intrinsic dynamics to the collective dynamics of the interactions with the teacher. Then the dynamical interaction process gives access to behavioral regions that the lone individual cannot reach by himself (Vygotsky, 1980; Froese and Fuchs, 2012). The learner is by now able to emancipate on his spontaneous tendencies by escaping their attraction. Thanks to the interactive participation of the teacher, he learns new patterns throughout the modification of his underlying dynamical landscape, expanding thereby his embodied musical personality.

## Conclusion

Personality (Kyselo and Tschacher, 2014), musical experiences (Dumas et al., 2014) and free collective improvisations (Canonne, 2012) are by now considered as embodied, dynamical, and social phenomena. Therefore, they can be linked theoretically. The Kaddouch pedagogy fits that framework (Laroche and Kaddouch, 2014). Learning by interacting during free improvisations enables to reveal embodied musical personality. This is done first by identifying the most stable, spontaneous achievement zones of the learner, and then by helping the learner to fulfill his core tastes, which can be masked by his spontaneous preferences.

By regulating the interaction process skillfully, and by using its collective dynamical properties, the teacher can orientate the learner's embodied behaviors and experiences, either by stabilizing or by altering the dynamical landscape of his embodiment. By interacting, the teacher directly participates in the process of learning. Indeed, thanks to this method, the learner can strengthen and expand his behavioral repertoire. He also learns to be flexible at reorganizing his behavior to interact skillfully with others; consequently he also learns how to share the creation of new performances. In this process of “participatory sense-making” (De Jaegher and Di Paolo, 2007), his behavior becomes meaningful from both his own perspective and the perspective of his interactions with the teacher. This is why learners prefer to fulfill their core tastes instead of accomplishing their sole spontaneous preferences. Since interactive improvisations and subsequent learning modify the dynamical landscape of a subject's embodiment, our method also reveals and expands musical personality. Therefore, interactivity is not only a way to probe embodied musical personality, but also a necessity to reveal and constitute it fully. In order to test this hypothesis further, we are currently working at quantifying and modeling the dynamics of performances, both at an individual and a collective level of analysis.

### Conflict of interest statement

The authors declare that the research was conducted in the absence of any commercial or financial relationships that could be construed as a potential conflict of interest.

## Footnotes

^1^We refer simultaneously to the whole experience that a subject has accumulated in the past, the experience he is currently living, and the one he anticipates or aims at living in the coming future.

^2^In this text, musical experience means “embodied experience of music.” This wording refers to all the experiences we have *with* music, that is, the experiences of “doing things with music” including listening to it (Krueger, 2011a).

^3^as evidenced by bodily and emotional reactions, and by his renewed motivation to explore further the improvisation when core tastes emerge in the interaction (Kaddouch and Laroche, 2014).
